# The Particle *Jako* (“Like”) in Spoken Czech: From Expressing Comparison to Mobilizing Affiliative Responses

**DOI:** 10.3389/fpsyg.2021.662115

**Published:** 2022-04-14

**Authors:** Florence Oloff

**Affiliations:** Languages and Literature, Faculty of Humanities, University of Oulu, Oulu, Finland

**Keywords:** conversation analysis, multimodal analysis, video data, spoken Czech, turn-final particles, mobilizing response, interactive turn space

## Abstract

This contribution investigates the use of the Czech particle *jako* (“like”/“as”) in naturally occurring conversations. Inspired by interactional research on unfinished or suspended utterances and on turn-final conjunctions and particles, the analysis aims to trace the possible development of *jako* from conjunction to a tag-like particle that can be exploited for mobilizing affiliative responses. Traditionally, *jako* has been described as conjunction used for comparing two elements or for providing a specification of a first element [“*X (is) like Y*”]. In spoken Czech, however, *jako* can be flexibly positioned within a speaking turn and does not seem to operate as a coordinating or hypotactic conjunction. As a result, prior studies have described *jako* as a polyfunctional particle. This article will try to shed light on the meaning of *jako* in spoken discourse by focusing on its apparent fuzzy or “filler” uses, i.e., when it is found in a mid-turn position in multi-unit turns and in the immediate vicinity of hesitations, pauses, and turn suspensions. Based on examples from mundane, video-recorded conversations and on a sequential and multimodal approach to social interaction, the analyses will first show that *jako* frequently frames discursive objects that co-participants should respond to. By using *jako* before a pause and concurrently adopting specific embodied displays, participants can more explicitly seek to mobilize responsive action. Moreover, as *jako* tends to cluster in multi-unit turns involving the formulation of subjective experience or stance, it can be shown to be specifically designed for mobilizing *affiliative* responses. Finally, it will be argued that the potential of *jako* to open up *interactive turn spaces* can be linked to the fundamental comparative semantics of the original conjunction.

## Introduction

The lexical item *jako* (English “like” or “as”) is highly frequent in spoken Czech. While *jako* has traditionally been described as hypotactic conjunction—that is, one that links two elements for comparison or provides additional information on a first element—and as a coordinating conjunction, most of its occurrences in spoken Czech do not seem to be covered by these traditional categories. Instead, *jako* in spoken discourse has been analyzed as a polyfunctional particle that signals, among other things, perturbations or hesitations in the process of speech production, reported speech or thoughts, new topics or inferences, and so on. It has, therefore, been mainly understood as a device for structuring unplanned oral discourse, and even as a parasitic “filler word.” This contribution suggests adopting a micro-analytic perspective on some prototypical oral uses of *jako* in naturally occurring, video-recorded conversations in order to shed light on the apparent contrast between the traditional (i.e., according to standard grammars) and actual (i.e., according to its occurrence in spoken discourse) contexts of use of the item. Based on the framework of conversation analysis and multimodal interaction analysis, this contribution will investigate some of the typical lexico-syntactical and audible environments of *jako*, namely, in the vicinity of hesitations, disfluencies, and pauses. At the same time, the analyses will consider its position within larger discursive activities, how possible recipients respond to its use, and the embodied conduct of both current speakers and their interlocutors. This investigation of *jako* in its fuller sequential and multimodal context aims at understanding its current use in relation to its original, comparative dimension—that is, establishing a comparative relation between two discursive elements. The analyses of different excerpts from the ordinary conversation will illustrate that this basic comparative meaning is a recognizable and essential feature of *jako*. Indeed, *jako* systematically projects a second element that does not necessarily have to be expressed, such that *jako* can be followed by turn suspensions or used in a turn-final position. In sequence-initial actions, it is by simultaneously projecting and withholding a second “next” discursive element that *jako* opens up a slot for co-participant responsive action, and, more specifically, for *affiliative* responsive action.

Previous work on *jako* has provided rather fuzzy functional descriptions and has disregarded the exact sequential and multimodal dimensions of the token (Section Descriptions of *jako* in Written and Spoken Czech). Although crosslinguistic research on quotatives draws an interesting parallel with the comparative dimension of *jako*, this has not yet been explored in terms of its supposed “filler” or “parasitic” usage. Within conversation analysis, phenomena such as hesitations, unfinished utterances, or turn suspensions have been shown to systematically relate to the maintenance or reestablishment of mutual attention and understanding. More specifically, research on pre-pausal conjunctions in other languages has illustrated how these can transform into particles that act on the scope of the next action (Section Hesitations, Turn-Suspensions, and “Incomplete” Turns in Social Interaction, and Their Response-Mobilizing Potential). Here, it will be argued that perturbations or hesitations in the vicinity of *jako* are not merely *flagged* by this item. *Jako* rather allows, through the specification of some previous discursive elements, for the anticipatory resolution of possible trouble (Section Indicating Uncertainty and Hesitation—or Projecting Precision?). More generally, *jako* appears at points in the conversation at which a specific responsive action from the side of the co-participant(s) has been made relevant. Indeed, together with embodied displays composed of resources such as gaze toward the recipient, stance-related facial expressions, gestures, and/or head nods, *jako* can be exploited to mobilize response by providing an extension of a previous opportunity for responsive action (e.g., in the form of response tokens or pre-emptive completions) (Section Creating a Slot for Responsive Action With *jako*). This can explain the proximity of *jako* to assessments, and, more generally, its clustering within conversational big packages that involve the formulation of self-disclosure, of a personal stance, or of a subjective report (including reported speech or thoughts). This shows that *jako* can be exploited to mobilize *affiliative* responses (Section Mobilizing Affiliative Responses With *jako*). Finally, it will be argued that the fundamental comparative dimension of *jako* is maintained throughout these different uses, albeit in a more abstract way (Section Discussion). *Jako* simultaneously refers to some previous element and projects a related but more specific “next.” This “next” can be another lexical item, but can also be a discursive element, such as a specific type of co-participant response. This basic semantic feature of *jako* can explain its overall flexible position and frequency within utterances and its specific suitability for use in turn- or unit-final position.

## Background

### Descriptions of *jako* in Written and Spoken Czech

This section is dedicated to the presentation of *jako* with respect to its basic grammatical descriptions and to prior research investigating its use in oral discourse. Due to its high frequency in spoken Czech, previous studies have aimed at distinguishing and classifying various functions of *jako*. While these inventories point at a rather heterogeneous set of contexts of use (oscillating between indicating precision/specification and, on the contrary, a lack thereof), research on *jako* as a quotation marker hints at a possible solution to this functional eclecticism: reconsidering *comparison* as a fundamental semantic feature of *jako* beyond its quotative uses.

In standard Czech dictionaries (Internetová Jazyková Příručka, Ústav Pro Jazyk Český, 2008–2021,^1^), *jako* is defined as a hypotactic conjunction used (a) for expressing a comparison and combining the elements that are to be compared (*velký jako já*, “as tall as me,” *zdravý jako ryba*, “healthy as a fish,” *je starš*í *jako matka*, “(s)he is older than mother”) and (b) for adding additional terms that contribute to the identification and illustration of a sentential element [*dojel jako prvn*í, “he finished (as) first,” *stěhovav*í *ptáci, jako vlaštovka a rehek, už odletěli*, “migratory birds, such as swallows and redstarts, have already flown away,” *takový člověk, jako je on, by to nikdy neudělal*, “a man like he is (i.e., like him) would never do that”]. Moreover, *jako* can be used as coordinating conjunction [*v zimě jako v létě*, “in winter like in summer” (i.e., “all year round”), *mlad*í *jako stař*í, “young and old (alike)”]. Its high frequency in spoken discourse^2^ (with more than 87,000 occurrences in the “oral_v1” corpus of the Czech National Corpus^3^; see also Adamovičová, 2017, p. 96, Table 1 and p. 98, Table 2), however, cannot be explained exclusively by these three traditionally described uses (comparative, illustrative, and coordinative). In spoken discourse, *jako* has been counted among the modifying particles, that is, tokens that contribute to the contextualization and communicative function of an utterance (“modifikační částice,” Nekula, 1995, p. 362–364), although *jako* is usually not explicitly acknowledged in its particle function in standard grammars (cf. Rinas, 2013, p. 174). Alternatively, *jako*, in spoken Czech, is treated as a discourse marker, as it is mainly used for utterance structuring and segmentation and only rarely according to its initial meaning, that is, for “comparing” and “explaining” (Čermáková et al., 2019, p. 316–318). Despite the fact that it is sometimes described as a “parasitic” item or the manifestation of somehow “deficient” speaking skills (cf. Nekula, 1996, p. 97–98; Michalec, 2017), other studies have attempted to systematize its occurrences in spoken discourse, the most important ones being those of Kolářová (1994) and Hoffmannová (2010).

Kolářová (1994) provides several empirical examples for the illustrative function of *jako* (explaining or specifying prior elements of the utterance). She notices that *jako* is often preceded by a pause “which signals that the speaker manifestly reflects on their wording, and often signals the vagueness of the speaker's idea of what they would like to express”^4^ (Kolářová, 1994, p. 168). According to the author, *jako* not only relates to the uncertainty of speakers about how to express specific facts, but also to more mundane formulation difficulties, as it often co-occurs with corrections, unfinished utterance parts, or other “disruptive” modifying particles such as *prostě*, “simply,” *teda*, “really,” or *tak*, “so” (Kolářová, 1994, p. 168). Hoffmannová (2010) states the difficulty in capturing the precise meaning of *jako* and proposes a list of its possible communicative uses based on selected examples from the Czech National Corpus. These relate to, but also go beyond, the basic meanings as presented in standard dictionaries (Hoffmannová, 2010, p. 361–363, my translation):

- a) paraphrasing, providing additional information;
- b) providing explanation, correction, or precision;
- c) introducing an additional argument or step;
- d) expressing an inference or result;
- e) introducing a new topic;
- f) introducing one's own or someone else's reported speech or thoughts;
- g) framing a quoted element from a specific context;
- h) indexing uncertainty, a search of an expression, or approximation;
- i) use as a universal filler word, signaling hesitation, and supporting the *ad-hoc* planification of unprepared speech;
- j) use as an empty utterance-final appendage.

It can be noted that, according to an inventory by Hoffmannová, the different functions of *jako* seem to relate both to providing more specific and precise information and to flagging imprecision and uncertainty. This apparent contradiction is, however, not attended to (but see the analysis of *jako* as a quotative marker in the next paragraphs). Moreover, the existing studies try to grasp the meanings of *jako* based on single decontextualized utterances in that they do not (or not systematically—see Čermáková et al., 2019, p. 316–318) consider how the turn containing *jako* is preceded or responded to within a given conversation. Additionally, no study has sought to reflect on the relationship between *jako* and larger action trajectories or possible multimodal resources co-occurring with its use. Up to now, its meaning within dialogic structures has only been marginally considered (for instance, in “challenging” questions, cf. Rinas, 2007, p. 399–401; 2013, p. 170–171, or relating to the formulation of inferences, cf. Hirschová, 2014, p. 91–93). However, these usages have been described based on written examples, and one can assume these to correspond to less frequent and more figurative uses of *jako*.

The only communicative function of *jako* that has received more detailed attention is its quotative use, although it does not seem to be the only lexical item used in Czech for framing reported speech and quotes (cf. Hoffmannová, 1999; Hoffmannová et al., 2017). According to Hoffmannová et al. (2017), the items *jako*, “like,” *teda*, “really,” and *jakože*, “as if” are used for indexing the reported speech of both the speaker and others and can occur alone or in combination with, for instance, *verba dicendi* (such as ří*kat*, “to say,” cf. Hoffmannová et al., 2017, p. 19). From a crosslinguistic perspective, *jako* is part of a group of quotative items deriving from a semantic source of comparison, that is, indexing similarity or approximation (Buchstaller and van Alphen, 2012, p. xiii–xv; see also Buchstaller, 2014, p. 20–22), which seems to be the most frequent semantic source overall in both Indo-European and typologically different languages. This relates to the natural impossibility of rendering reported speech in its verbatim original form, making the reported version by definition an approximation (or “demonstration,” cf. Clark and Gerrig, 1990) of the original. This kind of quotative thus enables the “[…] speakers [to] acknowledge and even highlight the approximative value of the quotation and thereby shield themselves from potential criticism regarding the inexact nature of the reproduction […]” (Buchstaller and van Alphen, 2012, p. xv). Comparative quotatives such as *jako* are therefore especially suitable for framing a specific stance, perspective, or opinion (with different degrees of certainty from the perspective of the speaker), and they index an exemplification of a given situation or turn-at-talk rather than its exact reproduction (see, e.g., for English: Romaine and Lange, 1991; Buchstaller, 2014; for French: Fleischman and Yaguello, 2004; for Hebrew: Maschler, 2002; for Norwegian: Hasund et al., 2012; cf. also the discussion on the “authenticity” of reported speech in Holt and Clift, 2007, p. 6–9). While traditionally, research on quotatives has more clearly focused on lexical and syntactical resources, a certain number of studies have also underlined the importance of other interactional resources, such as prosody, voice quality, or facial and other body movements, for framing and completing quotations of thoughts and (in)direct speech (e.g., Ferrara and Bell, 1995; Günthner, 1997; Golato, 2000; also see Keevallik, 2010 for how body movements themselves can become the quoted object). These bundles of vocal and embodied resources used in quotations often occur in the environment of story climaxes (Drew, 1998; Golato, 2000) and therefore allow other participants to affiliate with the depicted perspective or even to concurrently join the emerging utterance (Holt, 2000; Haakana, 2007; Selting, 2017). Several lines of reasoning ensue from these findings. First, the assumption of a possible basic meaning of *jako* with regard to its quotative use (e.g., “comparison”) may be fruitfully extended to its nonquotative uses as well. Second, the way *jako* is framed and accompanied by embodied actions might give more precise hints as to its interactional meaning. Finally, it might be useful to explore the link of *jako* to precise sequential and actional environments and how it relates to alignment and affiliation (Stivers, 2008).

Up to now, *jako* in Czech has been analyzed as having multiple communicative uses (or as being part of a larger heterogeneous group of items characteristic of spoken Czech, e.g., Hoffmannová, 1999, p. 71–83; Hoffmannová, 2018), some of which seem to possess a clear scope (e.g., the quotative use), while others appear to be rather fuzzy and underdetermined. The latter point is visible in the recurrent designation of *jako* as a “filler word” and as dispensable, and in the partial overlapping of different suggested uses (Hirschová, 2014, p. 89–90; Šulecová, 2015; Hoffmannová et al., 2017, p. 19; see also abovementioned categories a–j of Hoffmannová, 2010). As a result, the precise scope and meaning of various occurrences of *jako* in spoken Czech still remain to be tackled. Instead of suggesting a further functional differentiation, in this contribution, I will focus on instances of *jako* that, with regard to prior descriptions of this item, appear the fuzziest or underspecified. These instances relate to disturbances in the progression of the turn, that is, cases in which *jako* co-occurs with hesitations, cut-offs, or incomplete syntactic constructions (i.e., in turn- or unit-final position). They roughly correspond to the abovementioned categories of Hoffmannová (2010, p. 363), specifically (h) indexing uncertainty or a search, (i) indexing hesitation, and (j) acting as an “empty” utterance appendage. They thus include usages of *jako* with seemingly maximal fuzziness or semantical “emptiness.” In the analyses presented below, I intend to take into account the notion that comparison is an overall basic semantic feature of *jako* (cf. Pečený, 2009, p. 65–76) that can lead to a new perspective on the nonquotative uses of this item as well.

### Hesitations, Turn-Suspensions, and “Incomplete” Turns in Social Interaction and Their Response-Mobilizing Potential

This section will focus on two interrelated topics that are relevant to understanding the “fuzzy” occurrences of *jako* that are to be analyzed in this contribution. On the one hand, it will present a general outline of research in conversation analysis and multimodal interaction analysis on disfluencies, hesitations, and suspended or incomplete turns. On the other hand, it will look into research on turn-final conjunctions and particles in other languages. This will allow for a sketch of the lines along which the later analyses will be developed. Momentarily incomplete or suspended turns can mobilize responsive co-participant action, and former conjunctions in turn- or unit-final position have been shown to transform into particles that can specify the type or scope of the next action.

Instead of merely understanding disfluencies in typical speakers as the audible result of internal cognitive difficulties in language processing, numerous interactional studies have pointed out the systematic features and interactional meaning of recurrent “disfluencies” in social encounters. As Sacks et al.'s seminal work on the organization of turn-taking (Sacks et al., 1974) has shown, participants in social interaction can rely on various grammatical, lexical, and para-verbal resources in order to recognize when a given unit of talk—or turn-constructional unit (TCU)—has reached possible completion, thus enabling speaker-transition. The recognition of these transition-relevance places (TRPs), however, does not depend solely on the grammatical *completeness* of the turn. Indeed, knowledge about recurrent syntactical structures (Lerner, 1991, 1996) and grammatical projective force (Auer, 2005) allows co-participants to largely anticipate these points of possible completion, and also to continue an utterance on behalf of the original speaker. However, not only specific syntactical or actional structures (Lerner, 1991; Hayashi, 1999; Keevallik, 2013: p. 10–12), but also other interactional resources, such as pauses, cut-off words, or other hesitation phenomena (Lerner, 1996, p. 256–267), prosodic cues (Brenning, 2015), or the carrying out or retraction of specific gestures, movements, and object manipulations (Olsher, 2004; Mondada, 2006, 2015; Keevallik, 2020), contribute to the foreshadowing and formatting of moments of possible utterance completion. While co-participants can opportunistically self-select upon recognition of such points in the conversation, current speakers may also actively shape their utterances so as to invite the collaboration of a co-participant (Lerner, 2002; Kalkhoff and Dressel, 2019; Pfänder and Couper-Kuhlen, 2019).

Hitches and perturbations in an ongoing utterance have indeed been shown to trigger various types of responsive conduct in co-participants. The action of briefly suspending and restarting a turn-in-progress systematically leads a non-gazing recipient to reorient their gaze to the current speaker (Goodwin, 1981, 1995, p. 199–205). Whereas this type of perturbation of an utterance in progress aims at securing the attention of a recipient, more explicit or elaborate hesitation phenomena can also seek other types of recipient involvement. A display of uncertainty can be multimodally framed (e.g., by gazing toward a co-participant, not away from them, or by producing a “searching” gesture) as projecting a response from a knowing recipient (Goodwin, 1987), or securing help in retrieving a missing lexical item (Goodwin and Goodwin, 1986; Hayashi, 2003). Basically, the interpretation of a disruption in utterance progressivity as making relevant a response of a co-participant or not does not depend on grammatical structure alone but relies on a bundle of multimodal resources. For this reason, a speaker can, for instance, foreshadow a positive evaluation with a pre-positioned smile or by nodding, and thereby “guide” the recipient to producing an early positioned affiliative response (Goodwin and Goodwin, 1987), despite the actual assessment having not yet been formulated. Additionally, speakers can delay or withhold the production of a next unit-in-progress so as to provide an opportunity for the recipient to respond (Goodwin, 1986); that is, speakers can extend a prior TRP, for example, by producing an audible inbreath or some other non-lexical element. This enables the recipient to concurrently join with an affiliative response (e.g., an assessment) without intruding into the current speaker's next unit of talk.

Iwasaki's notion of “interactive turn spaces” (Iwasaki, 2009, 2013, 2018, inter alia) describes projective structures that do not operate *between* one TCU and another, but *inside* such a unit of talk. These local projective displays invite a responsive action or “micro-collaboration” from the co-participant, for instance in the form of continuers, response tokens, or pre-emptive completions (see also Hayashi, 2004). In Japanese, a noun phrase seems to be a recurrent grammatical resource for projecting such an interactive turn space if combined with prosodic (e.g., sound stretches or pauses) and embodied (e.g., gaze or nods) clues. Rather than simply providing a *possibility* for the recipient to respond (such as in the case of anticipatory completions), “[i]n contrast the suspended units […] constitute an invitation, or request, to the recipient to come in and produce a next relevant action so that the speaker can share and negotiate their stance” (Iwasaki, 2018, p. 72). While they do not work on the same structural level and have not been described for the same language, there are some similarities between the Japanese interactive turn spaces and “designedly incomplete utterances.” Particularly, the latter can be used in specific institutional settings to elicit information or demonstrations of knowledge from a co-participant (Koshik, 2002; Persson, 2017). Chevalier (2008) shows how syntactically unfinished utterances in French, due to their precise sequential positioning in pre-sequences, are followed by a pragmatically fitted response that does not treat the incompleteness of the previous turn as problematic. More specifically, unfinished turns seem to be frequently linked to potentially *delicate* social actions (Chevalier and Clift, 2008; Chevalier, 2009; see also Lerner, 2013; Li, 2016), and project, through both their sequential context and incompleteness, a preferred, *affiliative* response:

“[…] [Not completing a turn] is a resource that constitutes one way of addressing talk that is in some way delicate or problematic either in the development of the sequences or in the type of social actions that speakers seek to accomplish. In such a context, not completing a turn is one format deployed as a way of seeking affiliation.” (Chevalier and Clift, 2008, p. 1746)

Interactional research on hesitations and suspended or unfinished utterances shows us that, on the one hand, participants can exploit language-specific grammatical resources (cf. Stivers and Rossano, 2010) and other, translinguistically available embodied resources in order to elicit not *any* kind of responsive conduct, but more specific responses (such as affiliative ones) from their co-participants. On the other hand, these studies demonstrate that turn suspensions seem to be frequently associated with moments in which evaluations, stances, or possible delicate matters are dealt with. In the analyses presented below, I would like to argue that the occurrence of *jako* in spoken Czech often relates to interactional moments in which the response of an interlocutor has been made relevant, and that the response slot flagged by *jako* can aim at affiliative responses more particularly.

Studies on turn-final conjunctions and/or particles reveal themselves to be especially useful for understanding the apparent functional fuzziness of *jako*. In general, pre-pausal conjunctions (such as *and, but, or*) are ambiguous with regard to their implication for turn-taking, that is, they can be used as both turn-holding and turn-yielding devices (Jefferson, 1983; Mulder and Thompson, 2008). In her study on the Finnish conjunctions *ja*, “and” and *mutta*, “but,” Koivisto (2012) describes how these become recognizable turn-final *particles* when followed by a prosodic break or no further talk. Turns with turn-final *ja* can work as topic proffers (by providing incomplete/extendable lists) and thus mobilize a response, while turns with turn-final *mutta* can mitigate a potentially problematic stance (for instance, self-praise, or self-deprecation) and be used to pursue a response that is still due with respect to a prior turn (Koivisto, 2012, p. 1266–1269). The ambiguity of pre-pausal conjunctions remains, however, one of their inherent features, flexibly allowing for a continuation of the projected syntactic structure (“but > Y,” “and > Y”) in case no co-participant response is forthcoming: “For the participants, this ambiguity affords a possibility to transform the interpretation of the final conjunction ‘on the fly,' to accommodate the momentary interactional needs” (Koivisto, 2012, p. 1270).

A similar ambiguity with regard to turn-continuation or -yielding (ambiguous in the sense that the second element of a possible bipartite structure might be subsequently expressed by the same speaker or not) can be found in unit-final “or.” While “or” can be used as conjunction presenting two alternatives (“X or Y,” typically in alternative questions), it can also occur as a particle in turn-final position, that is, without actually expressing the alternative. This is the case of English *or* (Drake, 2015), German *oder* (Drake, 2016), and Finnish *vai* (Koivisto, 2017): these items can, for instance, transform a declarative turn into a confirmable, hint at an (unexpressed) alternative and therefore tilt the responsive preference away from an agreement with the first fully expressed alternative, or make relevant a topical elaboration by the second speaker. Thus, turn-final particles can create and adjust interactional spaces for responsive action. The type of responsive action targeted by these turn-final particles is usually reminiscent of the basic semantics of the initial conjunction (*or* and some of its lexical equivalents in other languages, for instance, act upon polar constraints, i.e., related to confirmation or disconfirmation as a relevant next action; cf. Drake, 2016). Consequently, *jako*, having been described both as conjunction and as a particle, might undergo a comparable process of transformation from conjunction to a pre-pausal or turn-final particle (cf. Mulder and Thompson, 2008). This could also provide an explanation for the previously mentioned apparent contradiction of *jako* signaling either precision or vagueness (cf. Section Descriptions of *jako* in Written and Spoken Czech). As the second element of the comparative relation set up by *jako* can but does not have to be systematically expressed, at least some instances of *jako* are very likely to mobilize a response. In order to reflect on this possible transformation process and the response-mobilizing potential of *jako*, a sequential and multimodal approach will be adopted in this contribution. Similar to the research that has been quoted in this section, I will consider the position of *jako* within its larger conversational context while simultaneously taking into account the precisely timed use of other audible and visible resources (cf. e.g., Goodwin, 1981; Streeck et al., 2011; Deppermann and Streeck, 2018; Mondada, 2018).

## Data Description

This study is based on video recordings of ordinary conversations among groups of two to four well-acquainted native speakers of Czech in private (i.e., at home) or public (i.e., cafés or bars) settings. The data was collected between 2013 and 2016^5^, with each recorded event lasting between 55 and 180 min. The recordings were based on the informed written consent of all involved participants, allowing for their use for scientific purposes. The participants' names, other proper names, and forms of personal information have been systematically replaced with pseudonyms or otherwise anonymized. The overall data set used for this contribution consists of 11 communication events with 19 different participants and with a total duration of nearly 14 h. Using raw transcripts (based on the transcription conventions as suggested by Jefferson, 2004, and Kaderka and Svobodová, 2006,^6^) and the original recordings, a first overview of the occurrences of *jako* has been established. While it was initially planned to investigate the full data set and the overall distribution of *jako* regarding its sequential position (e.g., turn-initial, mid-turn or -unit, or turn-final), the sheer number of its occurrences and the need to systematically verify the transcription and utterance segmentation led me to adopt a more exploratory approach at this stage instead. Despite different frequencies according to the data sets (between 46 and 169 instances of *jako* in the first 30 min of each conversation), *jako* is overall very frequent, with more than 1,000 cases in less than half of the data set (5.5 h). Moreover, this item has a tendency to appear in clusters, that is, with multiple occurrences in longer multi-unit turns by one speaker, such as storytelling or explanations. I, therefore, focused on a qualitative analysis of about 30 selected excerpts, each containing several occurrences of *jako* in the immediate vicinity of hesitations and turn suspensions in multi-unit turns. I then investigated the speakers' embodied conduct throughout the utterances containing *jako*, and, more specifically, their co-participants' possible responsive conduct with regard to the occurrence of *jako*—that is, mostly minimal responsive conduct such as head nods or response tokens. The excerpts presented in Section Results of this contribution do not represent a traditional collection in the conversation analytic sense (cf. Sidnell, 2012). Instead, they will serve to illustrate a line of reasoning that shows both the polyfunctionality and flexibility of this lexical item and its possible development from an intrasentential and supposedly monological item to an interactional response-mobilizing particle. The selected occurrences of *jako* have been multimodally annotated using the Mondada (2019) transcription conventions. Screenshots of the video recordings used in the analysis (Figures) are referred to with hashtags and continuous numbering (i.e., #1, #2, #3), and have been positioned in the transcript at the exact moment they were taken. In addition to an idiomatic line-by-line translation of the transcripts, simple glosses (following the Leipzig glossing rules^7^) have been provided for selected lines in the transcripts.

## Results

This section is dedicated to the presentation of some of the most frequent uses of *jako* in spoken Czech as they appear in the abovementioned data set. Due to its complex inflectional system, word order in Czech is quite flexible (see Oloff and Havlík, 2018), and as a particle, *jako* can be positioned at nearly any point within an utterance, as shown in examples 1–3 below^8^. Note that in Example 3, *jako* is positioned at the end of a (grammatically unfinished) turn and that another speaker will self-select immediately after *jako*.

**Example 1:**
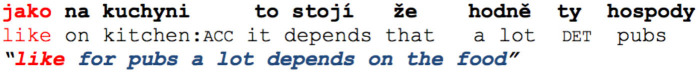
*jako* in turn-initial position.

**Example 2:**
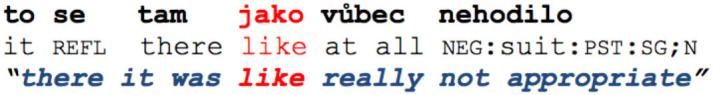
*jako* in mid-turn position.

**Example 3:**
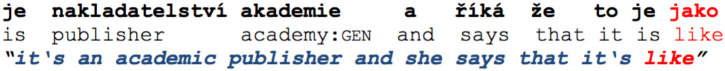
*jako* in turn-final position.

From these short and decontextualized examples, it appears that *jako* does not take on any specific grammatical or semantic role with respect to the meaning of the utterance (contrary to the written examples that illustrate its traditional functions; see the beginning of Section Descriptions of *jako* in Written and Spoken Czech). In what follows, I will demonstrate that even these apparently dispensable occurrences of *jako* are suited to specific types of social action. First, the connection between *jako* and perturbations in the production of an utterance will be studied (section Indicating Uncertainty and Hesitation—or Projecting Precision?). Then, it will be shown how, through the use of *jako*, speakers can create or extend a slot for the responsive actions of their co-participants (section Creating a Slot for Responsive Action With *jako*). Finally, it will be argued that *jako* frequently occurs in bigger conversational packages relating to subjective reports or stances, in which it is used for mobilizing affiliative responses (section Mobilizing Affiliative Responses With *jako*).

### Indicating Uncertainty and Hesitation—Or Projecting Precision?

Taking as an analytical starting point the understanding (as advanced by, e.g., Hoffmannová, 2010; Čermáková et al., 2019, p. 317–318) that *jako* is often related to the expression of uncertainty and hesitation or serves as an empty turn-final appendage, I suggest investigating different examples of *jako* in the vicinity of discursive perturbations without limiting the point of view to lexical content and syntactic structure. Instead, I will draw attention to non-lexical and other bodily resources co-occurring with *jako* or in its vicinity, and to the larger sequential context of its usage. By doing so, I wish to argue that *jako* does not a priori flag a word-search activity (i.e., it does not necessarily relate to the momentary cognitive unavailability of a specific word or expression from the point of view of the speaker). On the contrary, it projects an upcoming specification of some discursive element (in TCUs that are, for all practical purposes, not necessarily syntactically well-formed) in that it systematically provides for a specification of something previously stated or initiated.

*jako* is frequently positioned next to apparent perturbations in the production of speech, that is, hesitation particles, lexical fragments, and cut-offs, or pauses. However, there seems to be a distributional tendency: pauses are frequently positioned after *jako* (see Examples 6–7 below), while other types of perturbation instead cluster before *jako* (see Examples 4–5 below). Nevertheless, *jako* seems to mark the beginning of some specification or exemplification (cf. Examples 4–5) of what has preceded (see functions a–f of Hoffmannová, 2010; cf. section Descriptions of *jako* in Written and Spoken Czech), in the form of, for instance, a nominal phrase (Examples 4, 5), an adjectival phrase (Example 6), or even a complex sentence (Example 7).

**Examples 4–7:**
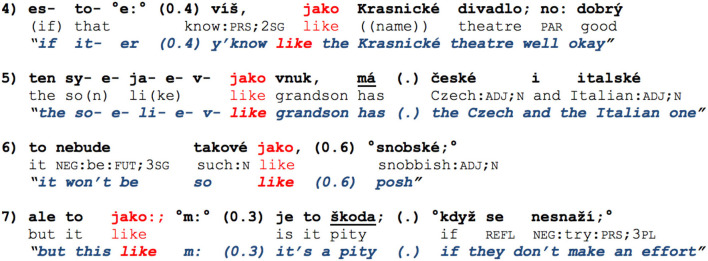
*jako* in the vicinity of utterance perturbations.

While the distribution in the examples given above—*jako* either preceded by audible perturbations or followed by a pause—could hint at a functional difference, the following analyses will demonstrate that a sequential and multimodal perspective on the same examples might lead to a more holistic understanding of what *jako* is used for in everyday communication. Rather than treating cut-off words or pauses as exclusively connected to a decontextualized speaker-related trouble, we can also relate these to the interactive management of recipiency and intersubjectivity (as has been masterfully shown in classic studies such as Goodwin, 1979, 1981; Schegloff, 1979; Goodwin C., 1980; Goodwin M. H., 1980; see also Drew, 1997; Schegloff, 2010, cf. section Hesitations, Turn-Suspensions, and “Incomplete” Turns in Social Interaction, and Their Response-Mobilizing Potential).

Let us first take a look at the larger sequential context of Example 4, which is now Example 8. The two friends Lucie (LUC) and Radka (RAD) are meeting at a café. At this point in the conversation, Lucie is talking about the latest play she attended, a piece by Havel, and now reports that she initially hesitated to attend it. This decision process and weighing of different arguments for and against going to the theater are multimodally formatted.

**Example 8:**
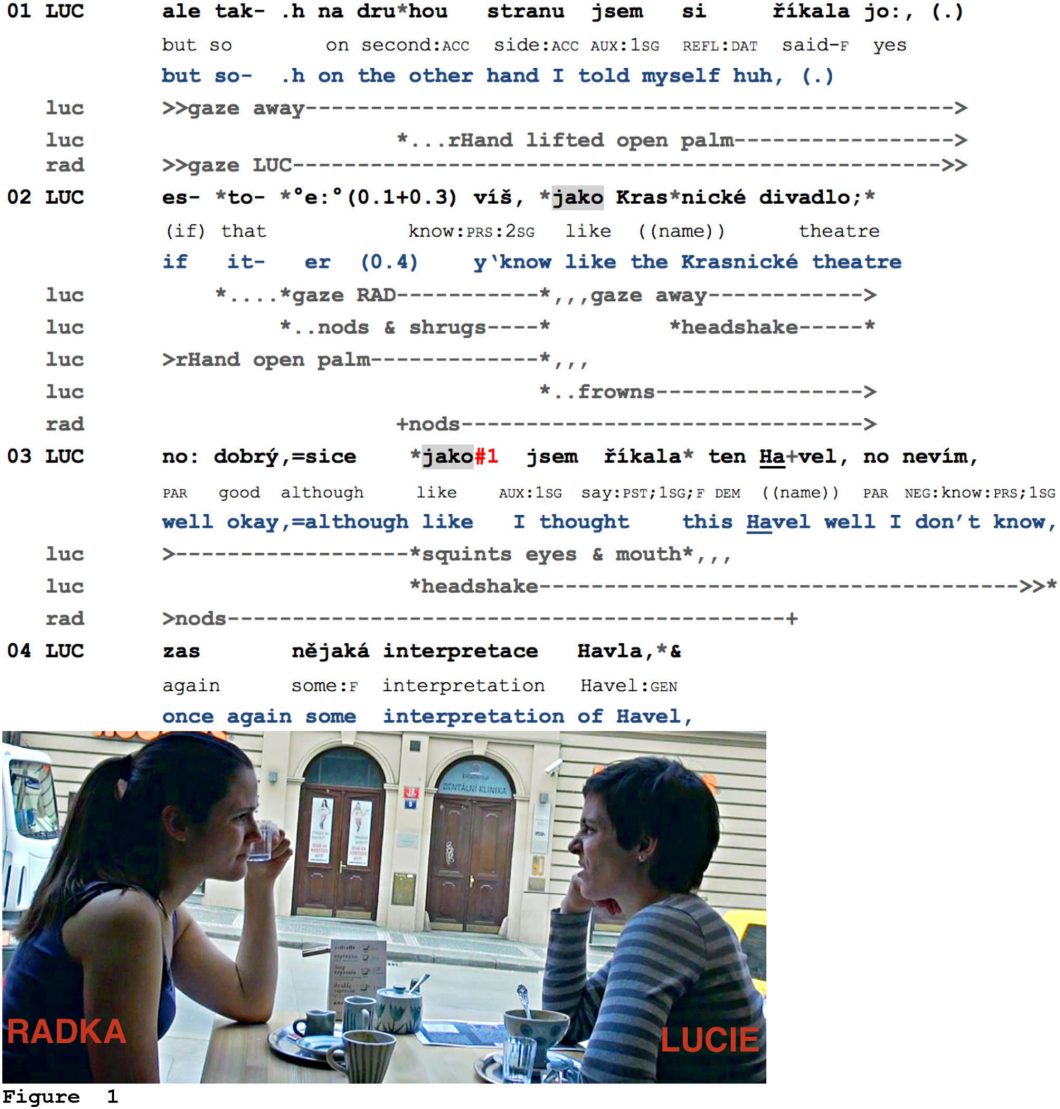
MamaCoff_003527_divadlo.

Lucie introduces a second argument here (“on the other hand,” l.01). While she seems to use *jako* for framing reported speech or thought (“I told myself,” l.01, “I thought,” l.03), it can be noticed that both instances of *jako* actually also frame an evaluation, first of the reputation of the theater, then of the author of the play. The multimodal annotation shows that preceding the first *jako*, Lucie holds up the palm of her right hand, nods, and shrugs, then, on the *jako*, starts frowning, and finally, while pronouncing the name of the theater, starts shaking her head. While the theater is being evaluated rather positively (“well okay,” l.03), the play is introduced contrastively by *sice*, “although.” Lucie again produces a headshake, but this time screws up both her eyes and mouth (Figure 1). This negative evaluation is then further formulated in the rest of her turn (i.e., the choice of play is not very original and thus it might not be worth attending, l.03–04). Interestingly, her interlocutor Radka, who is continuously gazing at her, accompanies the parts of the turn that are framed by *jako* with small head nods, thereby minimally responding to the offered assessments (l.02–03).

Although in the next example (previously Example 5), *jako* is not straightforwardly related to an assessment, it illustrates that this particle frames parts in the speaking turn that are relevant for responsive action from the interlocutor. Here, Jana (JAN) and her acquaintances, Nora (NOR) and Anna (ANN), discuss the possibilities for keeping or obtaining dual nationality for Czechs living abroad.

**Example 9:**
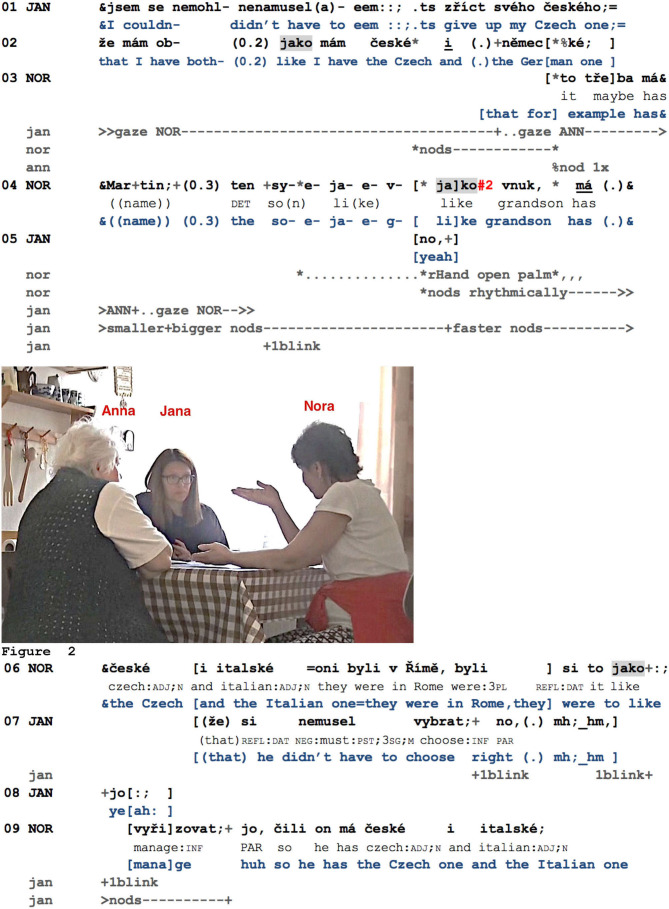
SOUSED_000524_obcanstvi.mov.

Jana, living with her family in Germany, informs her interlocutors that she did not have to yield her Czech nationality (l.01). She then formulates the inference, that is, that she, therefore, has both Czech and German nationality (l.02). Shortly after this inference introduced by *jako*, Nora nods in response, then self-selects in order to provide another example of dual nationality, namely, her grandson Martin, living with his parents in Italy (l.03–04). Using a recognitional (Schegloff, 1996) to introduce the grandson sets up its confirmation as a next relevant action. However, as Jana is still slightly nodding in order to manage Anna's visible response after her last turn (see the change of Jana's gaze orientation, l.02–04), this nodding does not provide a clear answer from Nora's perspective. Indeed, she does not proceed with her example but initiates self-repair regarding the proper name (*ten syn*, “the son,” then replaced by *vnuk*, “grandson,” l.04). Although Jana adapts her responsive conduct immediately by increasing the amplitude of her nodding, then by blinking (Hömke et al., 2017), Nora proceeds with her self-repair. This *jako* precedes the most suitable replacement for the proper name (*vnuk*) and thus marks the resumption of Nora's previously projected turn-construction. Concurrently with *jako*, Nora's open palm gesture toward Jana reaches its apex (Figure 2), and from this moment on, she accompanies her turn with rhythmical head nods. Jana upgrades her responsive conduct once again, now by using a response token (l.04–05; notice the exact overlap onset with Nora's *jako*) by increasing the tempo of her nodding and shortly afterward by formulating a candidate understanding in overlap, blinking once again and producing two more response tokens (l.07). Jana's next responsive conduct again coincides with Nora's second *jako*: Jana blinks twice and produces yet another response token (l.06–08): Jana blinks twice and produces yet another response token (l.06–08). It should be noted that this response token, *jo*, is positioned rather precisely in the small pause between the *jako* and the resumption of Nora's turn (the infinitive *vyřizovat*, “to manage,” l.09).

While it cannot be completely excluded that the pause between *jako* and the resumption of the TCU and turn in l.09 (also) relates to trouble in speech production or processing, there still seems to be a remarkable co-occurrence of the current speaker's *jako* and the recipient's responsive conduct. This can be further corroborated by excerpt 10 (previously Example 6), in which Lenka (LEN) visibly designs her turn for possible responsive action from Jana (JAN), who is sitting to her left. Beforehand, the participants were talking about different places to spend their holidays.

**Example 10:**
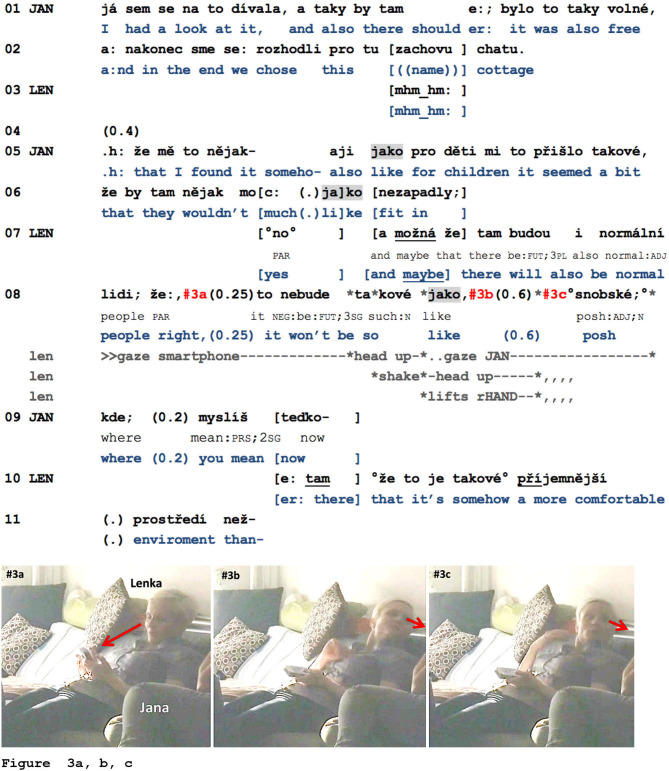
FLOW2_004158_snobske.mov.

Just before the beginning of the excerpt, Jana and Lenka have discussed a new and rather high-priced holiday resort. After having agreed that the high prices were related to the prestige and that this precisely attracted certain customers, Jana now states that she considered this resort first, but ended up booking a simpler cottage (l.01–02). While this turn reports on some factual action from her side and contains no *jako*, in her next turn, Jana accounts for her decision, and this personal evaluation (of the holiday resort not being suitable for children) is again framed by the use of *jako* (l.05–06; see also Lenka's responsive *no*, l.07). The more interesting occurrence of *jako* in this example is then produced by Lenka. She affiliates with Jana's account and thus her decision regarding the cottage by stating that “normal people” might also spend their holidays there (l.07–08). Jana does not seize the first opportunity to respond (see the TCU-final tag ž*e*, “right” and the following pause, l.08)—and Lenka then continues with a reformulated version of her previous statement. She also stops looking at her smartphone (cf. Figure 3a) and then, simultaneously with *jako*, enacts the yet-to-come adjective *snobské*, “posh”: she lifts her head, her chin, and her left hand so as to imitate a posture of “pride” (Figure 3b)^9^. She maintains this posture and looks at Jana throughout the following 0.6-s pause (cf. Figure 3c). As Jana is unfortunately excluded from the camera perspective, her embodied conduct cannot be analyzed; it is, however, certain that she does not formulate any audible response during this pause. Consequently, Lenka dissolves her posture and completes her turn with the adjective “posh.” That a response from Jana was indeed a relevant next action is shown by Jana's following repair-initiation and Lenka's repair (l.09–10). If one compares Jana's and Lenka's use of *jako* in this excerpt, the two previously described formats can be distinguished; on the one hand, perturbations followed by *jako* + phrase or clause, and on the other hand, no specific audible perturbations before the *jako*, followed by a pause and a resumption of the turn. The first three full examples (Examples 8–10) have illustrated that *jako* can indeed be used to provide more details and examples, that it often frames assessments, and that the discursive elements framed or accompanied by *jako* are treated as response-relevant by both the speakers and their recipients.

### Creating a Slot for Responsive Action With *jako*

This section will elaborate on the idea that *jako* opens up an opportunity space for co-participation. One of the affordances of *jako* is its flexibility with respect to its position within a syntactic structure; that is, as a particle it can be used *ad-hoc* at any moment in an emerging turn or TCU (cf. Examples 1–3). The downside of this positional flexibility is that, despite its capacity to project a slot for the co-participants' possible responsive action, *jako* might not be very precise with respect to the type of response that should follow. Indeed, by simply opening up a slot for a “next,” the exact *type* of projected next is then to be interpreted *in-situ* by the interlocutors (cf. section Hesitations, Turn-Suspensions, and “Incomplete” Turns in Social Interaction, and Their Response-Mobilizing Potential). Excerpts 11 and 12 will show two different types of responsive actions that co-participants might carry out in the post-positioned slot, namely, either response particles only or pre-emptive completions.

Preceding excerpt 11, Pavla (PAV, who is currently not in the room), Hana (HAN), and Jana (JAN) have been discussing the difficulty of staying slim beyond a certain age, and the fact that the absence of physical activity leads to an immediate weight gain. After Jana's complaint about this, Hana continues by relating her own experience regarding this issue.

**Example 11:**
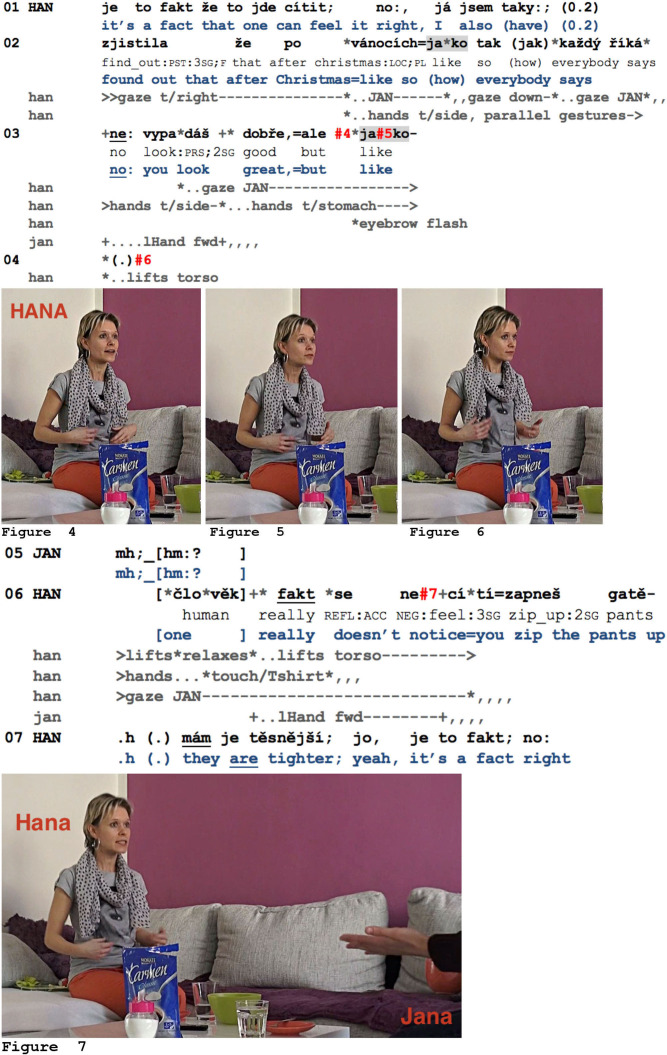
Pink1_000957_citit.mov.

Hana illustrates the contradiction between a slight but real weight gain and the fact that others do not necessarily perceive this (l.01–03). The first *jako* she uses (l.02) is actually related to the embodied completion (Olsher, 2004) of her TCU, embodying the noticing of her physical change: she first seeks Jana's gaze, then briefly positions her hands in front of her at the level of her stomach and simultaneously looks down, thus briefly pointing with both her head and hands to the body part that underwent a transformation “after Christmas.” In parallel, she continues her turn with a second observation—about others' perception of this change—looking back at Jana (cf. end of l.02). Note that this first *jako* thus completes a TCU, but it does not introduce the following reported speech here. Hana's second *jako* (together with *ale*, “but,” l.03) is latched onto the reported speech (“no you look great but like”) and initiates Hana's comment on this contradiction. Again, the emerging syntactical structure (starting with *ale*, “but”) is complemented by various embodied actions: Hana puts her hands closer to her stomach, and, precisely on *jako*, performs an eyebrow flash (see Figures 4–6). She then suspends her turn and immediately starts straightening her upper body, letting it go limp, straightening up again, and so on. This dynamic body movement visibly connects this *jako* to her turn resumption in l.06. Hana's visible assessment (cf. Goodwin and Goodwin, 1987) and her steady gaze to Jana strongly project a response from Jana. Jana had indeed already reacted to Hana's turn beginning by producing an affiliative gesture—a “palm addressed” gesture with her left hand in Hana's direction (cf. l.03)—thereby displaying her agreement (Kendon, 2004, p. 271–273). Before repeating this gesture (l.06), Jana produces a response token, which due to its lengthening and high, rising pitch is formatted as highly affiliative (cf. Figure 7; cf. Sørensen, 2021). It is well-timed with respect to Hana's turn suspension and embodied display. Hana then continues her turn by reformulating and transforming its initial part in a different order (“it's a fact”—“one can feel it”—then she provides a concrete example by pointing to her stomach, l.01–02—is transformed into “one really doesn't notice”—a concrete example, here, closing the zipper—and finally, “it's a fact,” l.06–07), these resembling statements flanking the opportunity space opened up by *jako*. Pausing right after a first assessment (here, *jako* + embodied display, l.03–04) also prevents Jana's affiliative responsive action from overlapping with too much of Hana's subsequent turn continuation (see Goodwin, 1986, p. 209–214).

The presence or type of a potential response slot, however, largely hinges on the way that *jako* is multimodally framed; thus, the response following *jako* can vary accordingly. Indeed, if hitches and perturbations also occur in its immediate vicinity, recipients might interpret the *jako* as being not primarily related to an assessment and to the projection of a response slot, but rather to an ongoing word search. The next example—with the same participants as in Example 11, but taken from a different encounter—shows that in such an ambiguous case, the co-participants can suggest a pre-emptive completion (instead of a response token only). Here, Jana reports on the previous evening, when she met up with Pavla and other former classmates at a bar. She complains about the waitress's unprofessional conduct, as the latter scolded the guests arriving one by one for ordering their drinks late. When the waitress showed up for the fourth time, Jana finally ordered “this bottle” (l.02), from which point the waitress finally stopped being rude. After having finished this report, Jana starts expressing her evaluation of the waitress's conduct (l.04–05).

**Example 12:**
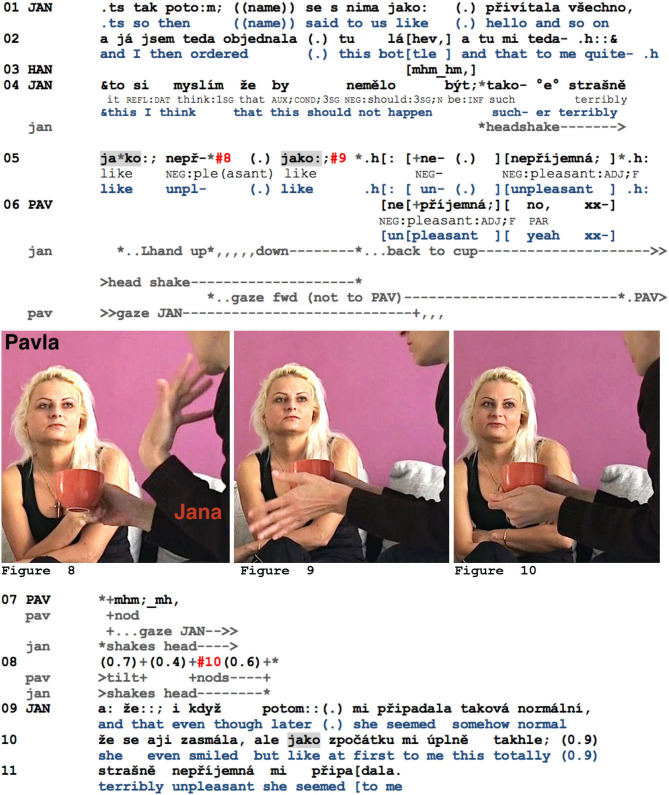
PINK2_0414_neprijemna.mov.

After a global and rather fluently formulated first evaluation (“this should not happen,” l.04), Jana seems to encounter some formulation difficulties (see also her abandoned construction at the end of l.02). The cut-off adjectives after the first and second *jako* in l.05, along with her baton gesture (with her left hand moving up and down, cf. Figures 8, 9; note that again, she is not fully visible in the video frame), hint at a possible formulation difficulty, although by shaking her head, Jana simultaneously indicates an emerging negative assessment (l.04–05). Pavla, who has continuously been monitoring her, now first offers the possibly sought-for adjective *nepř*í*jemná*, “unpleasant” (l.06). The fact that Jana carries out a full repeat of the suggested item also supports the understanding of the perturbations as possibly related to a word search (Oloff, 2014). But as Pavla then also adds a response token, she carries out a double responsive action, orienting both to the possible lack of a suitable lexical item *and* the presence of a responsive slot following *jako* (see also her supplementary response after Jana's repeat, l.07). However, it can be noted that—contrary to what happened in excerpt 11—Pavla's response may well be *aligned* but is not *affiliative* (cf. Stivers, 2008). She acknowledges Jana's negative assessment, but she does not explicitly agree with it (cf. also her gaze away from Jana in l.06, the absence of any specific prosodic emphasis on the acknowledgment token in l.07, and the absence of any affiliative facial expressions; cf. Figure 10). It is thus not surprising that Jana subsequently provides a reformulated version of her assessment (l.09–11), offering it once again for a possibly affiliative response. Note also that the 0.9-s pause in l.10 represents a slot in which Jana's co-participants could, but do not, self-select. Furthermore, the unfinished part of the syntactic construction introduced by *jako* projects a negative assessment of the waitress that is vocalized only after the pause (l.11). Audible and visible perturbations in the vicinity of *jako* can make it more difficult for co-participants to recognize the type of specific response projected by *jako*, such that instead of responding with a response token only, recipients might first provide a pre-emptive completion to the emerging turn instead.

### Mobilizing Affiliative Responses With *jako*

The last two excerpts will illustrate that *jako* can be utilized to create interactive opportunities for affiliative responsive action by extending a previously recognizable TRP. While this mobilizing of affiliative responses is not always successful, it can be systematically linked with specific key moments within bigger conversational packages. In excerpt 13, the four friends Anton (ANT), Milan (MIL), Karel (KAR), and Pavel (PAV) are having a drink after a joint amateur soccer match and are currently recalling their successful shots during today's match and previous ones. Karel now reports on a lucky streak in which he was prominently involved, having scored the second and third goal.

**Example 13:**
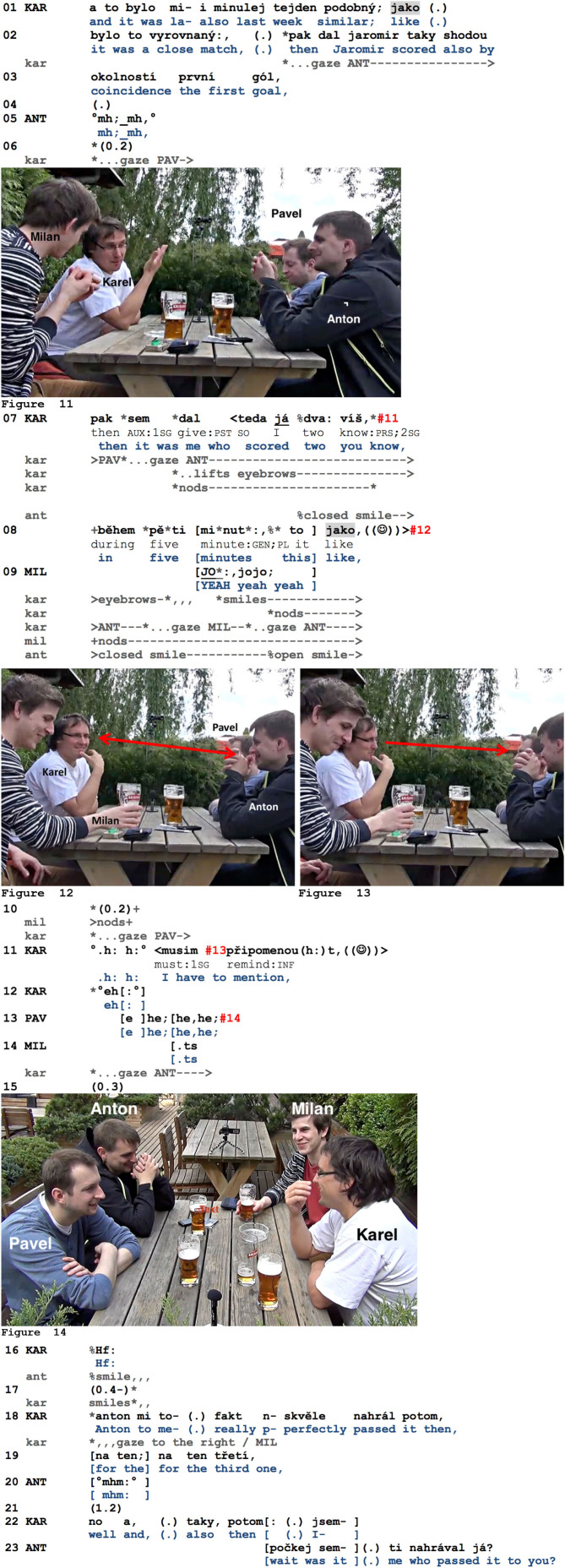
HAM_001119_dvagoly.mov.

Karel starts his sports report by explicitly drawing a parallel between two matches (the first *jako* in l.01, therefore, seems to relate to a comparison, and also opens up to a subjective report). Looking to Anton across the table, Karel then formulates what happened next, namely, that it was him scoring the next two goals (l.07). He multimodally frames this event as something positive and remarkable: while talking, he starts nodding, adopts a smiley voice, raises his eyebrows, and carries out an open palm gesture toward his co-participants (l.07, Figure 11). He then adds further “remarkable” information, specifically that it took him only 5 min to score these goals (l.08). During this continuation, he briefly reorients to Milan to his right and starts smiling. Milan is the first to produce an affiliative response, with a loud, lengthened, and then multiplied response token (l.09) and multiple head nods, thus participating in Karel's remembrance. Still in overlap, Karel begins the next unit with *to*, “this,” but suspends it soon thereafter. At this point, he visibly reorients to a next recipient who has not yet produced an audible response to his telling, Anton. Although Karel continues nodding and smiling after having suspended his emerging turn on *jako* (Figure 12), Anton does not seize this opportunity space to produce a verbal response, restricting himself to a more visible smile (precisely timed with the end of the previous TCU and thus clearly responsive). Not having obtained a more developed response from Anton, Karel now turns to Pavel (l.10, Figure 13), then produces some laughter particles, and resumes his suspended TCU (l.11, “this like (pause) I have to mention”), again with a smiley voice. Thus, a TCU suspended on *jako* can be resumed if the recipients fail to provide an appropriate uptake (cf. also Koivisto's analysis of some instances of turn-final *että*; Koivisto, 2014: p. 233–235, and section Hesitations, Turn-Suspensions, and “Incomplete” Turns in Social Interaction, and Their Response-Mobilizing Potential).

That Karel indeed seeks to mobilize affiliative responses from both Anton and Pavel can be seen in his subsequent conduct: he again reorients to Anton and produces some laughter particles, inviting the others to join (l.12, cf. Glenn, 2003). But Anton does not audibly join at this moment either, withdrawing his gaze from Karel and stopping to smile (l.16; note however that he keeps up his smile for the full length of this response opportunity slot). Pavel, on his part, apparently responds to the invitation to laugh (l.13), but without looking at Karel (cf. Figure 14), therefore producing an aligned but not affiliative response. Karel still tries to mobilize a possibly more developed uptake by producing yet another laughter token and continuously smiling (l.16–17). Not having obtained any further response, he continues his report, now giving more details on how he was able to realize one of these goals (l.18–19), namely thanks to Anton's pass. The fact that Anton rather mechanically and minimally acknowledges this detail (l.20), lets a long pause of 1.2 s pass (l.21), and finally—and in a late position—initiates repair regarding Karel's previous turn (l.23) demonstrates that he was not highly involved in Karel's telling at this moment. As a consequence, it can be stated that Karel's attempts to obtain affiliative responses have not been truly successful at this moment. However, this illustrates a large variety of practices for mobilizing affiliative responses: producing laughter particles, gazing at the respective co-participants one after another, incrementally producing different units that provide new details, and pausing in between in order to offer possible slots for responsive action. This clearly shows that a turn suspension on *jako* is not the only practice in Czech for generating response opportunities for co-participants. At the same time, it also illustrates the specificity of *jako*: it is the first practice to be implemented after an assessable has been offered to the participants. The fact that the turn construction is systematically resumed shows that it is specifically designed for providing a slot for responsive action within the current speaker's turn space—not afterward (cf. Iwasaki's interactive turn space, Iwasaki, 2009). The *jako* is therefore systematically accompanied by a full embodied display (here, smiling, nodding, and continuously gazing at a potential next recipient) that specifies the not yet formulated assessment, thereby giving the co-participants an extended opportunity to affiliate.

The fact that *jako* can be exploited to create and extend slots for affiliative action explains why it is especially frequent in moments in which speakers deliver some piece of subjective, personal, or even delicate news. In the last example, excerpt 14, Erika (ERI) is relating a difficult moment of her life to her friend Valerie (VAL). Lately, Erika has been watching a movie in which one of the characters dies slowly and painfully, triggering memories of her brother-in-law who passed away due to cancer. Here, Erika transforms the rather impersonal telling of the movie's storyline into a moment of self-disclosure, making relevant emphatic displays from Valerie's side (Kupetz, 2013). While reporting on the similarity of the movie to her relative's fate, Erika makes use of *jako* several times. In order to guarantee full anonymity to the participants in this specific case, no figures will be shown in the analysis.

**Example 14:**
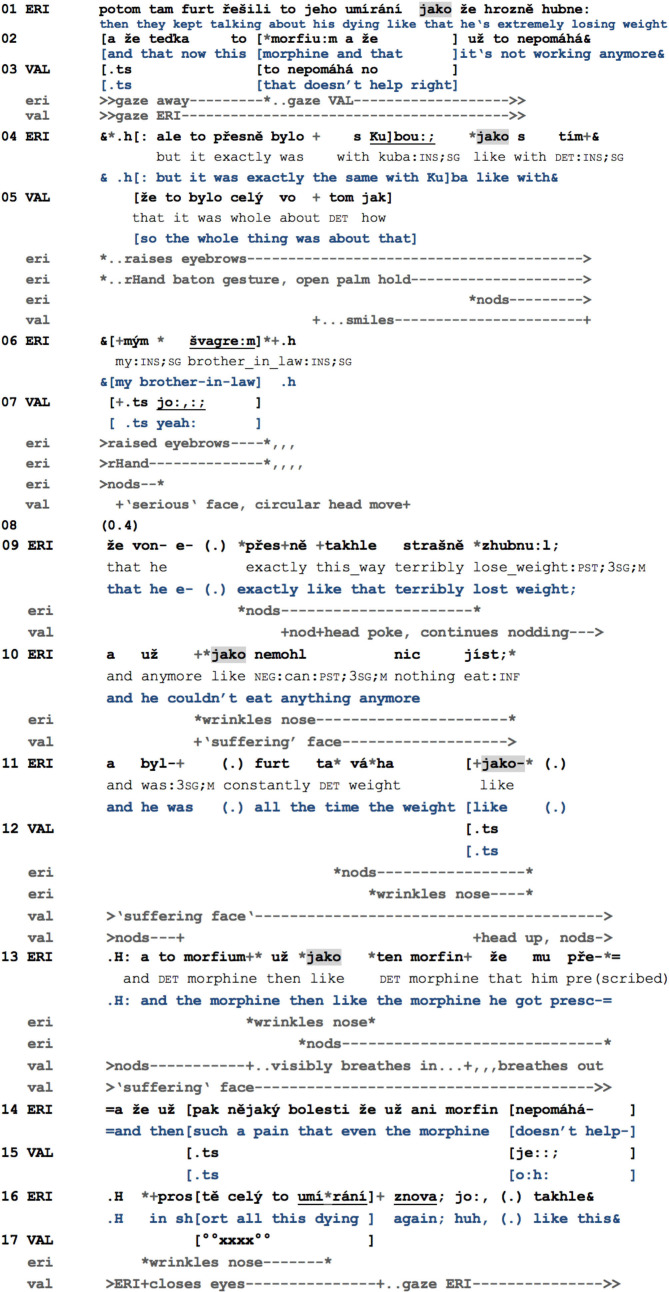
ANOCZ_005720_slzy.

Beforehand, Erika has already stated that the actor's performance did not captivate her. She now adds a further argument for why she did not like the movie, introducing its monotonous and rather depressing storyline in a three-part list (Jefferson, 1990) introduced by *jako*: weight loss, taking of strong medication, and the fact that in the end, the latter does not even help (l.01–02). While in the beginning, she is looking away, she turns her gaze to Valerie on the second item of the list (*morfium*), and both engage in mutual gaze until the end of the excerpt. Valerie responds to the first element of the list, referring and aligning to the overall negative opinion about the movie (l.03), also visible in her following remark on the “whole thing” (l.05), during which she even starts smiling. While Valerie is clearly pursuing the “neutral” topic, that is, the movie, Erika in the meantime is drawing a parallel with her personal experience (l.04). She now introduces her brother-in-law with his nickname, “Kuba.” Although she formats this part of her multi-unit turn as new and meaningful (raising her eyebrows and using an open palm gesture of her right hand; cf. Kendon, 2004, p. 265–271), Valerie does not display any recognition of the referent. Erika thus holds her gesture, and, similarly to what has been observed in excerpt 9, introduces a specification of the referent with *jako* (l.04, 06). During this turn, Valerie visibly grasps the new topical focus, as she suddenly stops smiling, adopts a serious expression, and, with a circular head movement, expresses her recognition of the referent and her understanding of the self-disclosing dimension of the talk (l.07).

In what follows (l.09–16), Erika recycles the list she established with respect to the movie (l.01–02), now applying it to the situation of her brother-in-law. Valerie affiliates with various and upgraded displays of empathy. Already during the first list item, the weight loss, Valerie starts nodding and then adopts a “suffering” facial expression simultaneously with Erika using *jako* for the second time (l.10). While weight loss in itself is neither specifically positive nor negative, the next description of Kuba's situation is indeed more serious (“he couldn't eat anything anymore”), which can be seen by Erika wrinkling her nose and contracting her eyebrows during this description. She adopts the same facial expression before and during the next *jako* (l.11). Despite this TCU being syntactically incomplete (“all the time the weight like”), Valerie upgrades her empathy display precisely in overlap with this *jako* by producing a lip smack, throwing back her head, and starting to nod again (l.12), which illustrates that *jako* indeed contributes to the extension of a responsive slot. Erika then recycles the second element of her former list: she introduces again the medication *morfium* and, using *jako*, starts specifying it (l.13). Once again, we can see that both participants simultaneously adapt their visible conduct at this moment, Erika by frowning and wrinkling her nose once again and Valerie by slowly and deeply breathing in. Only afterward does Valerie also respond audibly (by producing a response cry in l.15, a second step in the stepwise display of empathy, and then by more consistent turns in l.17; cf. Kupetz, 2013, p. 28). The summary of the telling (l.16, “in short all this dying again”) is again visibly framed, on Erika's side by the previously used wrinkling and frowning combination, on Valerie's side by closing her eyes during her mumbled utterance. The absence of *jako* in both this summary (l.16–17) and the beginning of the telling (see its first TCU, l.09) shows that *jako* does not seem to be used for openings and closings; that is, *jako*, in itself, does not project a possibly delicate “big package” (Jefferson, 1988) such as troubles-talk, complaints, narratives, or reports. However, it frequently occurs *within* such larger conversational units and is precisely positioned at moments in which the interlocutor's affiliative involvement has been made relevant, as shown by the coincidence of *jako* with affiliative responsive actions in this last example.

## Discussion

This contribution has aimed at investigating the use of the particle *jako*, “like” in naturally occurring Czech conversation. Both in publicly available corpora and in the dataset used in this article, the token *jako* was found to be extremely frequent. Its traditional function, as a conjunction that connects, compares, or states more precisely a pre-positioned discursive element, has been said to be marginal in spoken discourse. Previous corpus-based studies have described *jako* in spoken Czech as a polyfunctional particle, accomplishing various and different functions with respect to the initial conjunction: this particle seems to be able to operate both on the content of the utterance and on its structure. At the same time, most descriptions of its “communicative functions” hint at an apparent contradiction, as on the one hand, *jako* seems to introduce a specification or clarification (e.g., additional information, correction, result), while on the other hand, it seems to signal uncertainty and hesitation and has also been described as a “parasitic” or “filler” word. These eclectic and possibly contradictory functional categories underscore the need to investigate *jako* in spoken discourse in a more detailed way. Moreover, previous research has studied *jako* mainly on the basis of single decontextualized utterances, disregarding their larger sequential context, possible responses to these utterances, and the participants' overall visible conduct, all of which might contribute to a more thorough understanding of the pervasiveness of *jako* in social interaction.

The starting point of this analysis was to take a closer look at instances of *jako* in the vicinity of speech perturbations by adopting the analytical framework of multimodal interaction analysis. The data shows that *jako* does indeed frequently occur in the surroundings of utterance perturbations. But instead of automatically connecting *jako* to the existence of perturbations—for example, by viewing it as an actual signal of trouble regarding utterance progressivity—a closer look at some typical interactional environments illustrates that *jako* appears when a response by the interlocutor(s) has been made relevant, and, more specifically, that it is frequently connected to the delivery of subjective stances, decisions, or assessments. Indeed, the co-occurrence of *jako* with visible or audible responsive conduct by the current speaker's recipient is striking. While this response can be positioned in precise overlap with *jako* (indicating the recipient's recognition of a TRP), a specific practice demonstrates the use of this particle for actively *inviting* responsive conduct. In these cases, it is positioned at the end of a TCU (sometimes together with a fragment of a subsequent TCU), followed by a pause, and preceded or accompanied by embodied displays related to both assessment and uptake (such as nods, headshakes, evaluative facial expressions, and baton or open palm gestures). By combining *jako* with this type of embodied display, a participant appears to open up a slot for their co-participants in which responsive action can take place. During the pause, the recipients typically produce an affiliative response token, and the current speaker then resumes their previously suspended TCU.

This practice strongly resembles the interactive turn spaces described for Japanese interaction by Iwasaki (2009). Obviously, Czech and Japanese grammars do not provide the same grammatical resources for this type of practice, and I have suggested that *jako* has both specific advantages and disadvantages with respect to the mobilization of responsive action. On the one hand, due to its particle character, it can be positioned at any point in an emerging TCU, thereby providing a highly flexible resource for managing responsive conduct *ad-hoc*. On the other hand, *jako* itself does not provide specific grammatical information about the type of elements that are to follow. Moreover, on the semantic level, it merely projects a “next.” This means that—especially if it is positioned in the vicinity of utterance perturbations—co-participants might just as well interpret the action carried out by the current speaker as being a *word search*, and thus respond by suggesting a pre-emptive completion of the momentarily incomplete turn. Consequently, the way that *jako* is multimodally and lexically framed seems to be highly important, in that these resources can disambiguate which kind of responsive action should follow this particle. This could possibly be connected to its most frequent collocations, that is, *tak*, “so,” *ale*, “but,” *to*, “that,” *fakt*, “really,” *prostě*, “simply” or “just,” and *nějak*, “somehow” (Čermáková et al., 2019, p. 318); however, this would need to be investigated in a thorough qualitative study.

Another aspect that previous studies on *jako* did not clearly bring up is its link to specific types of conversational activities, such as stories, reports, and complaints. These big packages consist of multi-unit turns and usually involve a specific responsive involvement from the recipients' side. Once a current speaker obtains the go-ahead for such a conversational big package from their recipients, this makes relevant strategically placed displays of understanding, affiliation, or empathy. *jako* clusters remarkably within specific stretches of talk in a given conversation; that is, it is not distributed evenly or randomly, nor according to individual speakers (thereby refuting the “filler word” hypothesis). A closer look at these clusters shows that they are positioned within sequences in which a speaker elaborates on some personal event, decision process, complaint, or similar—that is, sequences in which the speaker is likely to disclose a personal assessment or stance at certain points, and to which the recipient should preferably affiliate. This connects with the general idea (corroborated in several crosslinguistic studies on incomplete utterances, pre-emptive completions, and pre-pausal or turn-final particles) that *jako*, in combination with a delay in turn progressivity, is a suitable lexical resource for mobilizing affiliative responses in potentially delicate environments. In order to verify this on a larger scale, one would have to systematically take into account quite a large lexical context for this token; that is, a more thorough quantitative study would first and foremost require an in-depth qualitative analysis of longer stretches of talk.

Now, how can the preceding observations be related to the initial function as conjunction and to the overall pervasiveness of the particle *jako* in spoken Czech? I would like to argue that this particle relies on the same semantic basis as the initial conjunction, that is, comparison, similarly to what has been shown for the quotative use of *jako* and comparable items in other languages. From traditional descriptions, it can be understood that *jako* basically establishes a relation of equivalence between two discursive elements, the first positioned before and the second after *jako*, i.e., X *jako* Y. The second element, Y, usually represents either a specification or another type of the first element, X. Consequently, once *jako* appears in an utterance, it marks the preceding elements as the first element of a comparative relation, simultaneously projecting a related second element, Y, that is yet to come. For this reason, *jako* usually flags an upcoming precision that aims at resolving a possible vagueness in the preceding parts of the utterance (and it can thus also *anticipate* other-initiations of repair, for example regarding the missing recognition of proper names). As *jako* projects an immediate next, its interactional scope is narrow rather than large, which in turn explains why it can be used repeatedly and in any position of an utterance or turn. Thus, its projective force is purely structural: *jako* can draw the recipient's attention to a preceding part of the utterance, thereby showing that what preceded is still relevant (even if the previous TCU has reached a TRP) and will be relevant for a second element to follow. This basic structural pattern seems to be exploited by Czech speakers in a more abstract sense as well. *Jako* does project a next, and if this next is not immediately delivered by the current speaker, this slot can be filled by the recipient, albeit with the constraint that the next is expected to directly relate to the first element. For this reason, *jako* is a highly suitable lexical resource for mobilizing aligned and affiliative responses in turn-final or unit-final position, especially if a first opportunity to respond (i.e., a preceding TRP) has not been immediately seized by the recipient. In that sense, *jako* can be used similarly to a tag, having transformed from an initial structure, “X *jako* > Y,” to “X *jako* > response to X.” More systematic analyses will have to be carried out in order to support this hypothesis and to tackle the precise role of embodied and other lexical resources in different occurrences of *jako* in natural conversation. More specifically, supplementary studies should aim to contrast the presence and absence of this particle in similar sequential environments (e.g., when is quotation introduced by items other than *jako*, and in which kind of multi-unit turns is *jako* used vs. not used?), and also consider, with respect to language change, whether certain types of uses or sequences involving *jako* will become more pervasive in the future.

## Data Availability Statement

The datasets presented in this article are not readily available due to ethical and privacy restrictions. Inquiries and requests to access the datasets should be directed to FO (florence.oloff@oulu.fi).

## Ethics Statement

Ethical review and approval was not required for the study on human participants in accordance with the local legislation and institutional requirements. The patients/participants provided their written informed consent to participate in this study. Written informed consent was obtained from the individual(s) for the publication of any potentially identifiable images or data included in this article.

## Author Contributions

FO organized and coordinated the original data collection and is responsible for the entirety of the manuscript.

## Funding

The data collection and preliminary analyses have been supported by the Swiss National Science Foundation (funding scheme Ambizione, project number 148146, 2014–2016). The publication fee for this contribution has been paid by the University of Oulu (Faculty of Humanities, Research Unit of Languages and Literature).

## Conflict of Interest

The author declares that the research was conducted in the absence of any commercial or financial relationships that could be construed as a potential conflict of interest.

## Publisher's Note

All claims expressed in this article are solely those of the author and do not necessarily represent those of their affiliated organizations, or those of the publisher, the editors and the reviewers. Any product that may be evaluated in this article, or claim that may be made by its manufacturer, is not guaranteed or endorsed by the publisher.

## Footnotes

^1^See https://prirucka.ujc.cas.cz/?slovo=jako (accessed December 15, 2021), including links to *jako*'s entries in two main Czech dictionaries.

^2^A simple search in the Czech National Corpus (https://www.korpus.cz/slovo-v-kostce/search/cs/jako, including both the default corpora “SYN2015” and “oral_v1”; accessed December 15, 2021) reveals 13,683 instances per million (IPM) words in spoken language, 4,324 IPM in fictional texts, 3,550 IPM in specialist literature texts, and 2,715 IPM in journalistic texts. According to Táborský (2010, p. 295), *jako* appears to be the most frequent particle in spoken Czech after *no*, “yes, well, right”.

^3^The oral_v1 corpus of the Czech National Corpus (cf. Kopřivová et al., 2017), a collection of mainly informal conversations collected between 2002 and 2011 from well-acquainted speakers from all regions of the Czech Republic, currently contains 6,361,707 tokens. For a thorough description of the oral_v1 corpus, please consult: https://wiki.korpus.cz/doku.php/en:cnk:oral (accessed December 15, 2021).

^4^My translation. Original quote: “[Často se slovo *jako* objevuje po pauze,] která naznačuje, že mluvčí zřejmě váhá nad formulací, a současně signalizuje neurčitost představy mluvčího o tom, co by chtěl vyjádřit.” (Kolářová, 1994, p. 168).

^5^This data was initially collected within the project “The epistemics of grammar: a comparative study of co-constructions in Czech, French, and German” (SNSF Ambizione funding, project number 148146, 2014–2016).

^6^In addition to the usual Jeffersonian signs for indicating intonation (. /, / ?), the semicolon (;) has been used to mark a slightly falling intonation (cf. Selting et al., 2009). The absence of any of the four aforementioned signs in TCU- or turn-final position implies level intonation (Jefferson, 2004, p. 27).

^7^The current version of the Leipzig glossing rules (revised version of February 2008, last changes on May 31, 2015) can be found here: https://www.eva.mpg.de/lingua/resources/glossing-rules.php (accessed December 15, 2021).

^8^In this sense, *jako* behaves quite similarly to other multiposition and multifunctional conjunctions and particles, such as Finnish *että*, “that” (Koivisto, 2014, p. 225; see also Laury and Seppänen, 2008, and section Hesitations, Turn-Suspensions, and “Incomplete” Turns in Social Interaction, and Their Response-Mobilizing Potential of this contribution).

^9^See also Keevallik's analysis (Keevallik, 2017) of Swedish and Estonian “but” in dance classes being accompanied by an embodied enactment. In her cases, the description and enactment of an incorrect dance movement is then followed by the conjunction “but” and the simultaneous or subsequent correct performance of the movement.
